# Nanostructured Photocatalysts and Their Applications in the Photocatalytic Transformation of Lignocellulosic Biomass: An Overview

**DOI:** 10.3390/ma2042228

**Published:** 2009-12-07

**Authors:** Juan Carlos Colmenares, Rafael Luque, Juan Manuel Campelo, Fernando Colmenares, Zbigniew Karpiński, Antonio Angel Romero

**Affiliations:** 1 Institute of Physical Chemistry, Polish Academy of Sciences, ul. Kasprzaka 44/52, 01-224 Warszawa, Poland; E-Mail: zk@ichf.edu.pl (Z.K.); 2 Departamento de Química Orgánica, Universidad de Córdoba, Edificio Marie Curie, Ctra Nnal IV, Km 396, E-14014, Córdoba, Spain; E-Mails: qo1capej@uco.es (J.M.C.); qo1rorea@uco.es (A.A.R.); 3 School of Engineering, Cranfield University, Cranfield, Bedfordshire, MK43 0AL, UK; E-Mail: r.f.colmenaresquintero@Cranfield.ac.uk (F.C.); 4 Gas Turbine Engineering Group, Faculty of Engineering, University of St. Bonaventure, Cra. 8H No. 172-20, Bogotá, D.C., Colombia

**Keywords:** heterogeneous photocatalysis, nanoparticles, biomass transformation, solar energy, biofuels

## Abstract

Heterogeneous photocatalysis offer many possibilities for finding appropiate environmentally friendly solutions for many of the the problems affecting our society (*i.e.*, energy issues). Researchers are still looking for novel routes to prepare solid photocatalysts able to transform solar into chemical energy more efficiently. In many developing countries, biomass is a major energy source, but currently such countries lack of the technology to sustainably obtain chemicals and/or fuels from it. The Roadmap for Biomass Technologies, authored by 26 leading experts from academia, industry, and government agencies, has predicted a gradual shift back to a carbohydrate-based economy. Biomass and biofuels appear to hold the key to satisfy the basic needs of our societies for the sustainable production of liquid fuels and high value-added chemicals without compromising the scenario of future generations. In this review, we aim to discuss various design routes for nanostructured photocatalytic solid materials in view of their applications in the selective transformation of lignocellulosic biomass to high value-added chemicals.

## 1. Introduction

**Scheme 1:**
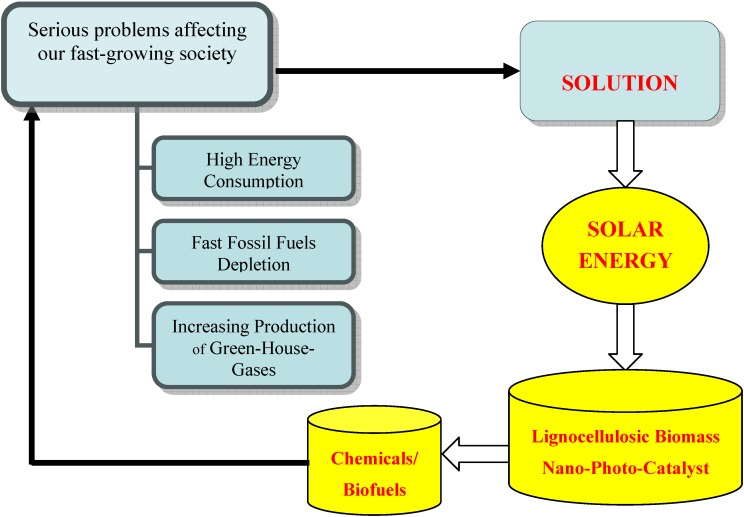
The efficient use of solar energy and biomass is considered a potential solution for energy and environmental challenges.

Nanotechnology has attracted a great deal of attention in the last few years as miniaturisation and nanomaterials are often foreseen to be the key for a sustainable future. In a broadest sense, nanochemistry makes use of the tools of synthetic and materials chemistry to generate nanomaterials with size, shape and surface properties that can be designed to evoke a specific function with the aim to be utilised in a particular application/end use. Nanotechnology allows us to manipulate the matter on a molecular scale (much less than 100 nanometers), helping us to obtain valuable information for the synthesis of new materials with specific properties and with a high degree of reproducibility. In this regard, an important part of the scientific community is currently focused on a very challenging and relevant research´s direction, which is the synthesis of novel nanostructured materials capable of absorbing the photonic energy coming from the sun with the aim of turning it into chemical or electrical energy (Scheme 1).

Many of these nanostructured materials find important applications in heterogeneous photocatalysis [1,2,3] due to the relevance of this multidisciplinary area as well as the multipurpose character of the solutions derived from it. Photocatalysis offers the possibility of extending the spectrum of applications to a variety of processes, including oxidations and oxidative cleavages, reductions, isomerizations, substitutions, condensations and polymerizations.

Applying the concept of nanotechnology to heterogeneous catalysis helps us understand more accurately the transformations ocurring on the catalyst´s surface at a molecular level [4]. The synthesis of materials with nanometric dimensions will facilitate a better understanding of the reaction mechanisms as well as to design novel useful catalytic systems. Nevertheless, despite several advances in designing new methods to obtain reproducible materials, there still exist numerous difficulties which need to be overcome. From the point of view of the materials, photocatalysts require a series of characteristic properties depending on their applications, including particle size, specific surface area or space between the electronic levels, among others.

Research activities have more recently focused on advanced oxidation processes (AOPs) for the destruction of synthetic organic species resistant to conventional methods. AOPs rely on the *in-situ* generation of highly reactive radical species, mainly HO•, by using solar, chemical or other forms of energy [5,6]. The most attractive feature of AOPs is that this highly effective and strongly oxidizing radical facilitates the degradation of a wide range of organic chemical substrates with no selectivity.

Heterogeneous photocatalysis involves the acceleration of photoreactions in presence of a semiconductor catalyst. One of the most relevant applications of heterogeneous catalysis is photocatalytic oxidation (PCO) to the partial or total mineralisation of gas/liquid-phase contaminants to benign substances [7].

Titania photocatalysis, also referred to as the “Honda–Fujishima effect”, was first revealed by the pioneering research of Fujishima and Honda [8]. These authors disclosed the possibility of water-splitting by means of a photoelectrochemical cell comprising of an inert cathode and a rutile titania anode. The application of titania photocatalysis was subsequently extended to environmental applications. Frank and Bard [9] reported for the first time the application of TiO_2_ in the photocatalytic oxidation of CN^−^ and SO_3_^2−^ in aqueous medium under sunlight. Further reports on the photocatalytic reduction of CO_2_ by Inoue *et al.* [10] attracted more interest in titania photocatalysis.

## 2. Basic Principles of Photocatalysis

Heterogeneous photocatalysis is a discipline which includes a large variety of reactions: organic synthesis, water splitting, photoreduction, hydrogen transfer, O_2_^18^–O_2_^16^ and deuterium–alkane isotopic exchange, metal deposition, disinfection and anti-cancer therapy, water detoxification, removal of gaseous pollutants, *etc.* [11,12]. Among them, titania-assisted heterogeneous photocatalytic oxidation has received more attention for many years as alternative method for purification of both air and water streams. The basic photophysical and photochemical principles underlying photocatalysis are already established and have been extensively reported [13,14].

A photocatalytic reaction is initiated when a photoexcited electron is promoted from the filled valence band of a semiconductor photocatalyst (SC) to the empty conduction band as the absorbed photon energy, *h*υ, equals or exceeds the band gap of the semiconductor photocatalyst, leaving behind a hole in the valence band. In concert, electron and hole pair (e^−^–h^+^) is generated. The following chain reactions have been widely accepted:

Photoexcitation:    TiO_2_/SC + *h*υ → e^−^ + h^+^ (1)

Oxygen ionosorption:    (O_2_)_ads_ + e^−^ → O_2_^•−^ (2)

Ionization of water:    H_2_O → OH^−^ + H^+^ (3)

Protonation of superoxides:   O_2_^•−^ + H^+^ → HOO• (4)

The hydroperoxyl radical formed in (4) has also scavenging properties similar to O_2_ thus doubly prolonging the lifetime of photohole:

HOO• + e^−^ → HO_2_^−^ (5)

HOO^−^ + H^+^ → H_2_O_2_ (6)

Both the oxidation and reduction can take place at the surface of the photoexcited semiconductor photocatalyst. Recombination between electron and hole occurs unless oxygen is available to scavenge the electrons to form superoxides (O_2_^•−^), its protonated form the hydroperoxyl radical (HO_2_•) and subsequently H_2_O_2_.

## 3. Mechanism of Titania-Assisted Photocatalysis

Titania has been widely used as a photocatalyst for generating charge carriers, thereby inducing reductive and oxidative processes, respectively [15]. Generally, Δ*G* is negative for titania-assisted aerobic photocatalytic reactions, as opposed to a photosynthetic reaction [11]. The corresponding acid A of the non-metal substituent is formed as by-product:

Organic wastes ⇒ TiO_2_/O_2_/ *h*υ≥E_g_ ⇒ Intermediate(s) ⇒ CO_2_ + H_2_O + A (7)

**Figure 1:**
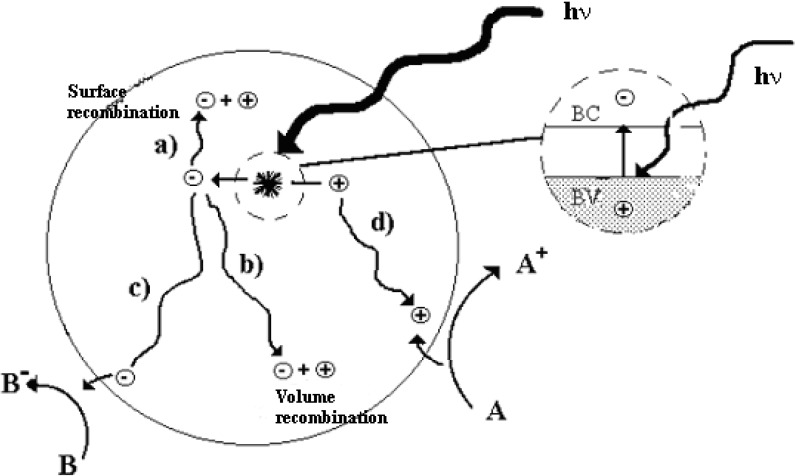
Photocatalytic process over TiO_2_. Adapted from Programa Iberoamericano de Ciencia y Tecnologia para el Desarrollo (*Red CYTED VIII-G).*

The [>Ti^IV^OH^•+^] and [>Ti^III^OH] represent the surface-trapped valence band electron and surface-trapped conduction band electrons, respectively. The surface-bound OH radical represented by [>Ti^IV^OH^•+^] is chemically equivalent to the surface-trapped hole allowing the use of the former and latter terms interchangeably [16]. According to Lawless and Serpone [17], the trapped hole and a surface-bound OH radical are indistinguishable species. A good correlation occurs between charge carrier dynamics, their surface densities and the efficiency of the photocatalytic degradation over TiO_2_. In the last two decades, aqueous suspensions of TiO_2_ have been probed by *pico*-second and more recently *femto*-second absorption spectroscopies [18,19]. Traditionally, an electron scavenger has been employed in such study. A *femto*-second spectroscopic study of TiO_2_/SCN^−^ aqueous system by Colombo and Bowman [18] indicated dramatic increase in the population of trapped charge carriers within the first few *pico*-seconds. The results also confirmed that for species adsorbed to TiO_2_, the hole-transfer reaction can successfully compete with the *pico*-second electron–hole recombination process. The following interfacial photochemical reactions were described (Figure 1):

Photoexcitation:   TiO_2_ + *h*υ → e^-^_CB_ + h^+^_VB_ (8)

Charge carrier trapping:    e^-^_CB_ → e^-^_TR_ (9)

Charge carrier trapping:    h^+^_VB_ → h^+^_TR_ (10)

Electron-hole recombination:  e^-^_TR_ + h^+^_VB_(h^+^_TR_) → e^-^_CB_ + heat (11)

Photoholes have great potential to oxidize organic species directly (although mechanism not proven conclusively [7]) or indirectly via the combination with OH• predominant in aqueous solution [13,20]:

H_2_O + h^+^ → OH• + H^+^ (12)

R–H + OH• → R• + H_2_O (13)

R• + h^+^ → R^+•^ → Degradation products (14)

Mediation of radical oxidative species in photooxidation was evidenced by photo- and electro-luminescence spectra of TiO_2_ electrodes in aqueous solutions measured as functions of the electrode potential and the solution pH [21]. It was found that the radical oxidative species originally absent accumulated after illumination under anodic bias. The primary photoreactions (1)–(11) indicate the critical role of charge carriers (electron–hole pair) in photooxidative degradation. Essentially, hydroxyl radicals (•OH), holes (h^+^), superoxide ions (O_2_^−^) and hydroperoxyl radicals (•OOH) are highly reactive intermediates that will act concomitantly to oxidize large variety of organic pollutants including volatile organic compounds (VOCs) and bioaerosols [22,23]. It is however argued experimentally that the oxidative reaction on titania photocatalyst surface occurs mainly via the formation of holes (with quantum yield of 5.7 × 10^−2^) not hydroxyl radicals formation (quantum yield 7 × 10^−5^) [24]. As a photochemical application, TiO_2_ photocatalysis is invariably affected by the surface properties of the TiO_2_ particle. The photoinduced phenomenon is affected by quantum size. Anpo *et al.* [25] observed a blue shift and increase in reaction yield and photocatalytic activity as the diameter of the TiO_2_ particles become smaller, especially below 10 nm. This observation was attributed to the suppression of radiationless transfer and the concurrent enhancement of the activities of the charge carriers.

## 4. Influence of Operational Parameters in Photocatalyst Efficiency

### 4.1. Light intensity

The photocatalytic reaction rate depends largely on the radiation absorption of the photocatalyst [26,27]. Recent reports revealed increase in the degradation rate with increase in light intensity during photocatalytic degradation [28,29]. The nature or form of the light does not affect the reaction pathway [30]. In other words, the band-gap sensitization mechanism does not have any significant influence in photocatalytic degradation. Unfortunately, only 5% of the total irradiated natural sunlight has sufficient energy to cause effective photosensitization [31]. Furthermore, energy losses due to light reflection, transmission and transformation into heat are inevitable in the photoprocess [32]. This limitation has greatly attracted more researchers to the applications of TiO_2_ to decontamination/detoxification. The overall quanta of light absorbed by any photocatalyst or reactant is given by Ø_overall_, the quantum yield:

Ø_overall_ = rate of reaction / rate of absorption of radiation

where the rate of reaction (mol/time) accounts for moles of reactant/s consumed or product formed in the bulk phase and the rate of absorption of radiation (Einstein/time) relates to the amount (*i.e.*, mol or einstein) of photons at wavelength *λ* absorbed by the photocatalyst.

The light scattering in solid–liquid regimes is particularly significant. Quantum yield is thus experimentally difficult to determine as metal oxides in a heterogeneous regime including TiO_2_ cannot absorb all the incident radiation due to refraction [33].

Another factor which limits photonic efficiency is the thermal recombination between electrons and holes [34]. For these reasons, it is argued that references to quantum yield or efficiency in heterogeneous system are not advised despite previous use of the term by previous references [35,36]. A practical and simple alternative for comparing process efficiencies was suggested by defining the so-called *relative photonic efficiency* **ζr** [37]. A quantum yield can be subsequently determined from ζr, as:

Ø = ζr Ø_compound_,

where Ø_compound_ is the quantum yield for the photocatalyzed oxidative disappearance of this chemical using a photocatalyst (e.g., Degussa P-25 TiO_2_ ).

### 4.2. Nature and concentration of the substrate

Organic molecules which can effectively adsorb to the surface of the photocatalyst will be more susceptible to direct oxidation [38]. Thus, the photocatalytic degradation of aromatics depends on the substituent group. Nitrophenol has been reported to be a much stronger adsorbing substrate than phenol and therefore degrades faster [39]. In the degradation of chloroaromatics, Hugul *et al.* demonstrated that monochlorinated phenol degrades faster than di- or tri-chlorinated derivatives [40]. In general, molecules with electron-withdrawing groups including nitrobenzene and benzoic acid have been found to significantly adsorb in the dark compared to those with electron-donating groups [41].

The concentration of organic substrates in time is also dependent on photonic efficiency during photocatalytic oxidation [42]. At high-substrate concentrations, however, the photonic efficiency decreases and the titanium dioxide surface becomes saturated, thus leading to catalyst deactivation [43].

### 4.3. Nature of the photocatalyst

There is direct correlation between the surface of organic reagents and surface coverage of TiO_2_ photocatalyst [44]. Kogo *et al.* reported that the number of photons hitting the photocatalyst actually controls the rate of the reaction [45]. The latter is an indication that the reaction takes place only in the adsorbed phase of the semiconductor particle. A very important parameter influencing the performance of nanomaterials in photocatalytic oxidation is the surface morphology, namely particle and agglomerate size [46]. Numerous forms of TiO_2_ have been synthesized by different methods with the aim to achieve materials exhibiting desirable physical properties, activity and stability for photocatalytic application [47]. Evidently, there is a clear connection between the surface properties, the rational development of improved synthesis routes and the potential usefulness of the material prepared for particular applications [48,49]. For instance, smaller nano-particle sizes have been reported to give higher activities in gaseous phase photomineralisation of organic compounds employing nanostructured titanium dioxide [50].

### 4.4. Concentration of photocatalyst

The rate of photocatalytic reaction is strongly influenced by the photocatalyst concentration, as expected. Heterogeneous photocatalytic reactions are known to show proportional increase in photodegradation with increasing catalyst loadings [51]. Generally, in any given photocatalytic application, the optimum catalyst concentration must be determined, in order to avoid excess catalyst and ensure total absorption of efficient photons [52]. This is due to the observation of unfavourable light scattering and reduction of light penetration into the solution with an excess of photocatalyst [53].

### 4.5. pH

The pH of the solution is an important parameter in reactions taking place on particulate surfaces as it controls the surface charge properties of the photocatalyst and size of the formed aggregates [54]. In the current update of the points of zero charge (pzc, it describes the condition when the electrical charge density on a surface is zero) by Kosmulski, Degussa P-25 (80% anatase and 20% rutile) is reported to have pzc 6.9 [55]. The surface of titania can be protonated or deprotonated under acidic or alkaline conditions, respectively, according to the following reactions:

TiOH + H^+^ → TiOH_2_^+^ (15)

TiOH + OH^−^ → TiO^−^ + H_2_O (16)

Thus, a titania surface will remain positively charged in acidic medium (pH < 6.9) and negatively charged in alkaline medium (pH > 6.9). Titanium dioxide is reported to have higher oxidizing activity at lower pH, but excess H^+^ at very low pH can decrease reaction rates [56].

The effect of pH on the photocatalytic reactions of organic compounds and adsorption on TiO_2_ surfaces has been extensively studied [57,58]. Changes in pH can result in enhancement of the efficiency of photoremoval of organic pollutants in presence of titanium dioxide without affecting the rate equation [59]. Improved degradation of such compounds has been reported under optimized conditions [60].

### 4.6. Reaction temperature

Experimental studies on the dependence of the reaction rate with temperature in the degradation of organic compounds have been carried out since 1970s [61]. Many researchers established experimental evidences for the dependence of photocatalytic activity with temperature [62,63,64,65,66]. Generally, the increase in temperature enhances recombination of charge carriers and desorption process of adsorbed reactant species, resulting in a decrease of photocatalytic activity. These facts are in good agreement with the Arrhenius equation, for which the apparent first order rate constant Ln*k*_app_ should increase linearly with exp(−1/*T*).

## 5. Properties and Characteristics of Photocatalysts: Titania *vs* Other Photocatalysts

An ideal photocatalyst for photocatalytic oxidation is characterized by the following attributes [11]:

(1) Photo-stability.
(2) Chemically and biologically inert nature.
(3) Availability and low cost.
(4) Capability to adsorb reactants under efficient photonic activation (*h*υ ≥ *E*_g_).

Titania is the most widely employed (nano)material in photocatalytic processes, although there are several (nano)materials currently considered as photocatalysts and/or supports for photocatalysis aside from titania. These include related metal oxides, metal chalcogenides, zeolites (as supports), *etc.*

### 5.1. Titania (TiO_2_)

**Figure 2:**
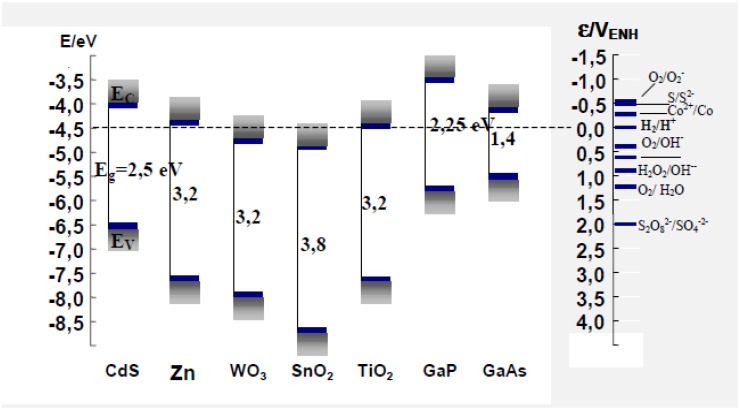
Diagram of conduction and valence band for various semiconductors. Adapted from Programa Iberoamericano de Ciencia y Tecnologia para el Desarrollo *(Red CYTED VIII-G).*

Nanometric size titania is by far the most widely employed system in photocatalysis due to its comparatively higher photocatalytic activity, low toxicity, chemical stability and very low cost. The anatase form of titania is reported to give the best combination of photoactivity and photostability [7]. Nearly all studies have focused on the crystalline forms of titania, namely anatase and rutile. The minimum band gap energy required for photon to cause photogeneration of charge carriers over TiO_2_ semiconductor (anatase form) is 3.2 eV corresponding to a wavelength of 388 nm [67]. Practically, TiO_2_ photoactivation takes place in the range of 300–388 nm. The photoinduced transfer of electrons ocurring with adsorbed species on semiconductor photocatalysts depends on the band-edge position of the semiconductor and the redox potentials of the adsorbates [20]. The schematic diagram of band positions for various semiconductors is shown in Figure 2.

#### 5.1.1. Types of TiO_2_ catalysts

The most frequently used TiO_2_ photocatalyst is the Degussa P25 material [68,69,70,71]. Its particle size is about 25 nm and its surface area is very small (50 m^2^/g). Reducing the particle size, up to a few nanometres, has the benefit of increasing the external surface area. These small particles tend to agglomerate by strong interparticle forces when the nanometric size region is reached. Further decrease of the particle size to a few nanometers reaches one point below which quantum size effects also start to operate and the band gap of the semiconductor increases, blue-shifting the light absorption.

Titania in the form of photocatalyst titania materials of 1D dimensionality such as nanotubes, nanofibers and nanowires have also attracted attention for their use in photocatalysis [72,73,74,75]. In particular titania nanotubes with 10–100 nm in diameter and micrometric length have been the subject of intensive investigations. Compared to spherical particles, one-dimensional TiO_2_ nanostructures could provide a high surface area and a high interfacial charge transfer rate. The carriers are free to move throughout the length of these nanostructures, which is expected to reduce the e^−^/h^+^ recombination probability. Titania nanotubes have a relatively high surface area compared to non-porous titania and time-resolved diffuse-reflectance spectroscopy has shown that charge recombination is disfavored by the tubular morphology of the titania. These titania nanotubes can be conveniently obtained starting from titania nanoparticles as, for instance P-25, that are digested under strong basic conditions in an autoclave at about 150 °C for several hours [76]. Annealing of these nanotube-TiO_2_ at 400 °C for 3 h rendered nanotubes that are composed 100% by anatase. Laser flash photolysis of the nanotube-TiO_2_ compared with conventional titania nanoparticles has allowed to estimate apparent quantum yield of charge separation (Ø_cs_). Table 1 lists some of the Ø_cs_ values measured for nanotube-TiO_2_ compared with different conventional spherical nanoparticles powders.

**Table 1:** Surface area and photophysical data obtained by laser flash photolysis of nanotube-TiO_2_ and other TiO_2_ materials. Reproduced by permission of the Royal Society of Chemistry from reference 77.

TiO_2_^a^	BET surface area/m^2^g^-1^	Ø_cs_	T_1/2_/µs
nanotube-TiO_2_-400	225	2.0	3.5 + 0.4
Standard TiO_2_-1	300	7.1	0.6 + 0.2
Standard TiO_2_-2	50	4.8	1.0 + 0.2
Standard TiO_2_-3	10	1.6	0.7 + 0.2

^a^TiO_2_—commercial samples of spherical shaped nanoparticles.

Despite the research efforts in the search for novel photocatalysts over the last two decades, titania (in its anatase form) has remained a benchmark to compare with any emerging material candidate [78]. Zhang and Maggard also reported the preparation of hydrated form of amorphous titania with wider band energy gap than anatase and significant photocatalytic activity [79].

There are important drawbacks that severely limit the application of titanium dioxide photocatalyst as a general tool either to degrade organic pollutants in the gas or liquid phase or to perform useful transformations of organic compounds [80,81,82,83,84,85,86,87]. One of the most important limitations is the lack of TiO_2_ photocatalytic activity with visible light [80,88,89] The reason for this is that the anatase form of TiO_2_ is a wide band gap semiconductor with a bandgap of 3.2 eV in most media, corresponding to an onset of the optical absorption band at about 350 nm. This onset of the TiO_2_ absorption is also inadequate to achieve efficient solar light photocatalytic activity, since approx. 5% of the solar light energy can be absorbed by TiO_2_. The above comments explain the continued interest in improving the photocatalytic efficiency of TiO_2_.

#### 5.1.2. Modified Titania systems for improved photocatalytic activity under visible light

Two general strategies have been developed to increase the photocatalytic activity of TiO_2_ for visible light irradiation, namely the use of an organic dye as photosensitizer or doping TiO_2_ with metallic and non-metallic elements [2,86,88,90,91,92,93,94,95,96,97,98,99,100].

The first route (using an organic dye that absorbs visible light) has worked very well under conditions where oxygen/air is excluded and the degradation of the dye is minimized by the efficient quenching of the dye oxidation state with an appropriate electrolyte [86,92,93,94,101,102,103,104,105]. Otherwise, particularly the dye becomes rapidly mineralized and the photocatalytic system loses its response towards visible light in the presence of oxygen. The mechanism of TiO_2_ dye sensitization has been determined using time resolved subnanosecond laser flash photolysis techniques [101,102,103,104,106,107]. In dye sensitization, the most relevant points are the absorption spectrum of the dye in the visible region and the energy of the electron in the excited electronic state of the dye, which has to be high enough to be transferred to the semiconductor conduction band.

A second chemical route to promote titania photoresponse into the visible spectra is related to the doping of TiO_2_ material either with metallic or non metallic elements [86,88,90,91,92,93,94]. In this case, doping introduces occupied or unoccupied orbitals in the band gap region leading to negative or positive doping, respectively. A summary of some novel preparations of UV and visible light responsive titania photocatalysts developed over the last few years has been recently compiled [108].

##### Doping with metals

Pt doping of titania has recently attracted a great deal of attention due to promising improvement in photooxidation rate especially in gas phase. Pt–TiO_2_ has been found to improve the photooxidation rate of ethanol, acetaldehyde and acetone in the gaseous phase [109,110]. A further development of mesoporous titania as photocatalyst has been reported by Li *et al.* [111]. These authors have prepared a photocatalyst constituted by mesoporous titania embedding gold nanoparticles (Au/TiO_2_). Its preparation requires P-123 as structure directing agent, a mixture of TiCl_4_ and Ti(OBu)_4_ and AuCl_3_ as the source of gold using ethanol as solvent. The gel is cast on a Petri dish to form a thin layer that is subsequently aged at 100°C to form a homogeneous mesostructured nanocomposite. Calcination at 350 °C in air removes the template while inducing crystallization of TiO_2_ and formation of gold nanoparticles.

TiO_2_ has also been doped with other metals, including V, Cr, Mn, Fe and Ni. The presence of the dopant was found to cause large shift in the absorption band of titanium dioxide towards the visible region. However, there are contradictory reports, particularly in the case of metal doping, describing either an increase or a decrease of the photocatalytic activity [2,12,97,112]. This controversy arises in part from the difficulty to establish valid comparisons between the photocatalytic activity of various solids testing different probe molecules and employing inconsistent parameters. Also, the doping procedure and the nature of the resulting material is very often not well defined and, most probably, controversial results can be obtained depending on the way in which the metal has been introduced. It also depends on the final concentration of the dopant. Thus, it has often been reported that there is an optimum doping level to achieve the maximum efficiency and beyond this point a decrease in photocatalytic activity is again observed [95,113]. Nevertheless, a generalised consensus has been reached with regards to the inappropriateness of metal doping as valid solution to enhance the photocatalytic activity of TiO_2_.

##### Doping with non-metallic elements

Doping with carbon, nitrogen, sulfur and other non-metallic elements has been recently reported to introduce visible light absorption on titanium dioxide [88,90,113,114,115,116,117,118,119,120,121,122,123]. Asahi *et al.* were the first to show an absorption increase in the visible region upon nitrogen doping [114]. This opened the way to study titania doping with non-metallic elements [98,115,124]. However, due to corrosion and instability of doped materials, it remains to be seen whether non-metallic element doping can be regarded as a general and valid approach to increase the photocatalytic efficiency of titania.

The photophysical mechanism of doped TiO_2_ is not yet understood but the p-type metal ion dopants (with valencies lower than that of Ti^4+^) are believed to act as acceptor centres as opposed to the p-type [80].

### 5.2. Binary metal oxides

In addition to TiO_2_, there are some other traditional metal oxides, which have also been extensively investigated due to their specific advantages. Many metal oxide semiconductors, including ZnO, ZrO_2_, Fe_2_O_3_ and WO_3_, have been examined and used as photocatalysts for the degradation of organic contaminants [125]. Among them, ZnO, α-Fe_2_O_3_ and WO_3_ are representative. However, they all have inherent drawbacks to be employed in photocatalysis. ZnO is easily photo-corroded under bandgap irradiation by photogenerated holes. WO_3_ is a stable photocatalyst for O_2_ evolution under visible light irradiation, but not suitable, for instance, for H_2_ production due to its low conduction band level. α-Fe_2_O_3_ has the same problem as WO_3_ and, moreover, is not very stable in acidic solutions.

A nanocrystalline mesoporous Ta_2_O_5_ photocatalyst for H_2_ production was recently synthesized through a combined sol–gel process with a surfactant-assisted templating mechanism [126]. The effects of NiO as co-catalyst loading and doping with Fe have also been studied [126,127].

### 5.3. Metal sulfides

Metal sulfides are normally considered attractive candidates for visible light responsive photocatalysis. The valence band of metal sulfides normally consist of 3p orbitals of S, which result in a more occupied valence band and narrower bandgap as compared to metal oxides. Recent studies have focused on CdS, ZnS and their solid solutions.

CdS has a suitable bandgap (2.4 eV) and good band positions for visible light assisted water splitting. However, S^2−^ in CdS is easily oxidized by photogenerated holes, which is accompanied by the release of Cd^2+^ into solution, similar to ZnO. Such photo-corrosion is in fact a common problem to most metal sulfide photocatalysts.

Recent developments aiming at improving CdS and ZnS photocatalysts can be divided into four directions: (1) synthesis of one-dimensional and porous CdS; (2) doping and formation of solid solutions of CdS and ZnS; (3) addition of co-catalysts to CdS; and (4) development of support and matrix structures for CdS.

**Figure 3:**
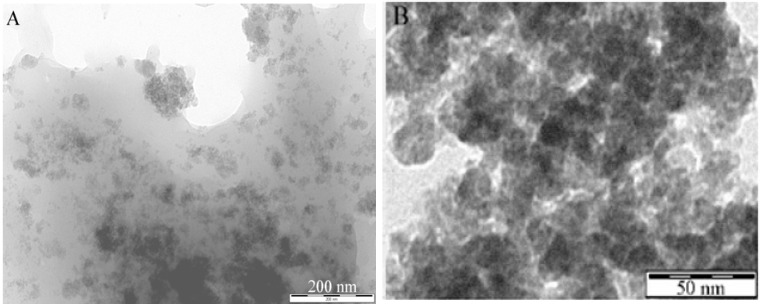
Mesoporous polysaccharide supported CdS quantum dots. Adapted from reference [132].

With regards to the synthesis of porous CdS, a solvothermal method was utilised to prepare CdS nanorods [128] and nanowires [129]. Mesoporous CdS nanoparticles with an average pore size of 5.4 nm and a particle size of 4–6 nm have also been prepared by template-free, ultrasonic-mediated precipitation at room temperature [130]. Bao *et al.* have also prepared nanoporous CdS, including nanosheets and hollow nanorods, by means of a two-step aqueous route, consisting of an initial precipitation of nanoporous Cd(OH)_2_ intermediates and a subsequent S^2−^/OH^−^ ion exchange [131]. The obtained CdS nanostructures contain pores with 3 nm diameter. White *et al.* have also recently prepared CdS quantum dots supported on porous polysaccharides as novel contrast agents to provide better insights into the pore structure of materials that cannot be seen by simple microscopy imaging [132] (Figure 3).

Comparatively, ZnS possesses a bandgap too large to respond to visible light (3.6 eV). Doping and formation of solid solutions of ZnS and narrow bandgap semiconductors can enhance the visible light utilization of ZnS. ZnS and CdS have similar crystal structures, which make them form solid solutions easily.

(AgIn)_X_Zn_2(1−X)_S_2_ solid solutions between ZnS and AgInS_2_ have a narrower bandgap. The absorption of the solid solutions shifted monotonically to longer wavelengths as the ratio of AgInS_2_ to ZnS increased. Photophysical and photocatalytic properties of these nanomaterials were highly dependent on composition mainly due to a change in band position caused by the contribution of the Ag 4d and S 3p, and Zn 4s4p and In 5s5p orbitals to the valence and conduction bands, respectively [133].

## 6. Preparation of Photocatalysts

### 6.1. Sol-gel method: A promising route for TiO_2_ nanophotocatalysts synthesis

The sol–gel process is currently considered one of the most promising alternatives due to its inherent advantages including low sintering temperature, versatility of processing and homogeneity at molecular level. This method allows the preparation of TiO_2_-anatase at low temperature. This phase has been extensively investigated because of its high activity in photocatalytic applications [84,134].

TiO_2_ powders and gels with porous structure and high photocatalytic performance have been reported [135,136]. However, the preparation of porous TiO_2_ films with high specific surface area is attracting more and more attention [137,138,139]. Photocatalytic processes are chemical reactions on the surface. The increase of surface area should improve the efficiency of the process as it implies larger contact surfaces exposed to the reagents [140,141]. Porous inorganic TiO_2_-anatase films can be obtained using templating membranes [142] or conventional alkoxide sol–gel route with the addition of surfactants [143]. Templates facilitate the retention of the initial polymer morphology up to the final porous structure. Polyethylene glycol is particularly suitable for modifying the porous structure of coatings [144,145] due to its complete decomposition at relatively low temperatures [146,147]. However, precise control on synthesis and deposition processes is crucial to achieve thick, crack-free and homogeneous coatings.

One of the advantages of sol-gel synthesis of mesoporous materials is the possibility to form uniform films on a substrate. Using the method studied in detail by Sanchez *et al.* [148,149] uniform films of mesoporous titania on glass can be obtained dipping the glass slide into an acidic solution of titanium alcoxide in ethanol. The surfactant concentration is lower than the critical micellar concentration (cmc) immediately after dipping the glass, but cmc is reached when the glass is gradually removed from solution (together with ethanol evaporation) and the surfactant starts to template the formation of thin layers of a mesoporous titanium oxide. The key point is to carefully control the rate of solvent evaporation to be sufficiently slow to allow the templation and oligomerization of the titanium oxide around the self assembled micelles created by the surfactant in its liquid crystal state.

**Figure 4:**
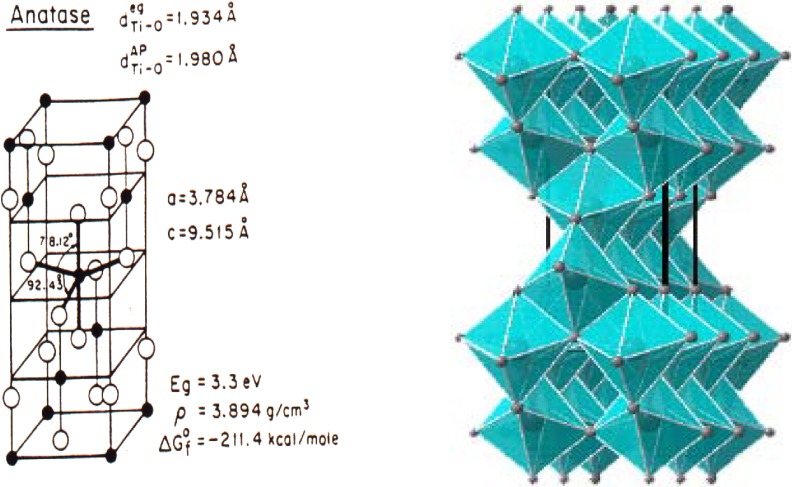
Structure of anatase.

Applying the above methodology, Stucky *et al.* prepared highly structured materials constituted by anatase nanoparticles (5–10 nm) ordered forming a mesoporous film of TiO_2_ perpendicular to the glass slide [150]. The as-synthesized TiO_2_ material is initially structured in the 5–100 nm length scale forming mesopores, but the walls are formed by an amorphous TiO_2_ phase. Calcination of the material produces crystallization of the as-synthetized amorphous titanium dioxide into anatase phase (Figure 4) without destroying the mesoporous ordering of the film. Careful control of the calcination temperature (<500 °C) is also crucial to avoid the formation of the significantly less photocatalytic active rutile phase.

### 6.2. Ultrasonic preparation of nanostructured photocatalysts

The use of non-conventional irradiation methods (e.g., sonication and microwave radiation) during the synthesis could be of help in order to avoid or at least reduce crystallite growth. In this sense, the use of ultrasonic irradiation during the synthetic procedure has been reported to facilitate the formation of smaller homogeneous nanoparticles and lead to an increase in surface area [2,151,152]

The presence of solid particles (metal supports) in the liquid system enhances the cavitation phenomenon under ultrasonic treatment [153,154] as the microbubbles tend to break up into smaller ones thus increasing the total number of regions of high temperature and pressure. Ultrasound can also enhance the mass transfer towards the liquid–solid interface [155].

Aging under sonication led to pure-anatase nanoparticles (the most active photocatalytic phase of titania) irrespective of the titanium precursor used and increased significantly the surface area of the nanostructured material. This enhancement in surface area resulted in an increase in molar conversion in the selective oxy-dehydrogenation of 2-propanol to acetone using Pt/TiO_2_ as a photocatalyst [2].

In the same type of reaction, the subsequent photodeposition of platinum on Ti- and V-containing zeolites results in a sharp increase in molar conversion, low or negligible deactivation with time-on-stream and significant increase in selectivity to acetone (90–96%) [156].

### 6.3. Other non-conventional synthesis methodologies

Different methods have been used to prepare TiO_2_ materials: reactive method [157], chemical vapour deposition, sputtering, pulsed laser deposition (PLD) [158] or hydrothermal method [159]. Mergel *et al.* [157] deposited films by electron beam evaporation of granular TiO_2_ of purity 99.5% in a BAK640 high vacuum chamber pumped with a diffusion pump. The thickness of the films obtained by this method ranged from 0.9 to 2.4 mm.

Yamamoto *et al.* [158] synthesized TiO_2_ films with anatase and rutile structure by pulsed laser deposition (PLD) with a NdYAG laser under controlled O_2_ atmosphere. The same authors have also successfully prepared epitaxial anatase films on several types of oxide substrates with different lattice parameters (LaAlO_3_, SrTiO_3_, MgO and yttria-stabilized zirconia; YSZ). The high quality epitaxial rutile films were also grown on α-Al_2_O_3_ substrate. During deposition, substrates were maintained at 500 °C and exposed to 35 mtorr O_2_ gas pressure. The typical thicknesses of epitaxial films were in the 200 to 880 nm range. From optical absorption measurements, the optical band gaps for anatase and rutile TiO_2_ epitaxial films were evaluated to be 3.22 eV and 3.03 eV, respectively. The contribution of photoactivated electrons and holes to photocatalysis in these epitaxial TiO_2_ films can be improved via further approaches to reduce crystal defects.

## 7. Photochemical Transformations of Biomass via Heterogeneous Photocatalysis

Photocatalysis is a good example of Green Chemistry. The relationship between Photocatalysis and Green Chemistry can be described in different ways. The name of the discipline itself is related to two of the so-called principles of Green Chemistry. “Photo” means light and if it is sunlight, as this is the case, there will be Energy Economy (6^th^ Principle). On the other hand, catalytic processes are always preferable to non-catalytic ones (9^th^ Principle). Furthermore, photocatalysis employed for the transformation of biomass has impact on another two Green Chemistry principles, the use of renewable feedstocks (7^th^ Principle) and the design for degradation (10^th^ Principle).

Lignocellulosic substances (e.g., wood) undergo UV-induced degradative reactions. Early studies from Stillings and Van Nostrand on cellulose showed cotton fibers irradiated with UV-light under nitrogen atmosphere underwent photochemical transformations that led to an increase in the number of reducing sugars with a corresponding evolution of carbon monoxide and carbon dioxide [159].

Desai and Shields also studied the photochemical degradation of cellulose filter paper 25 years later [160]. Working with rubber-stoppered static irradiation tubes that were initially filled with air (in contrast with the nitrogen-purged system of Stillings and Van Nostrand), they observed a spectrum of fully reduced and oxygenated hydrocarbons produced upon degradation. Such hydrocarbons were only observed after irradiation periods longer than 1–2 h, whereas no delay was observed in an initially oxygen-free atmosphere *(vide infra)*. Biomass, the most versatile renewable resource, could therefore be turned into a wide range of chemicals and derivatives by means of photocatalysis.

In this section, we will highlight some of the most trendy and high potential processes for future development in the photochemical transformations of a range of biomass using heterogeneous photocatalysts.

### 7.1. Photocatalytic hydrogen production

**Figure 5:**
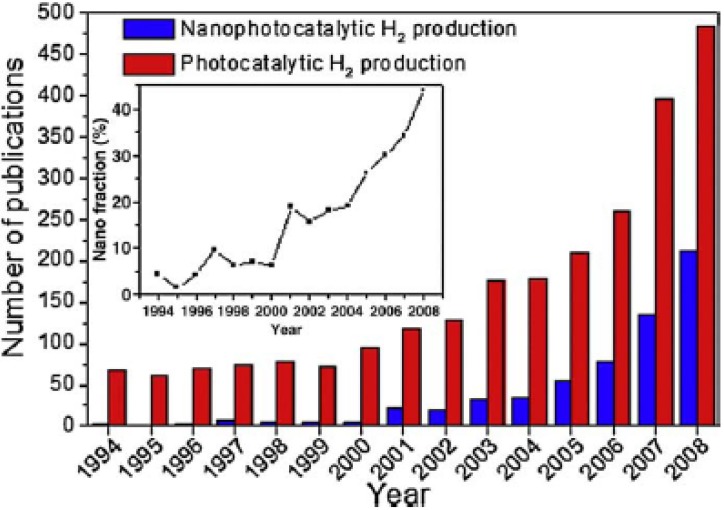
Evolution of the number of publications on (nano)photocatalytic production of hydrogen. Data from Web of Science (ISI, 2009).

Nanotechnology have boosted the modification of existing photocatalysts for the production of hydrogen and the discovery and development of new candidate materials, as shown in Figure 5. The rapidly increasing number of scientific publications on nanophotocatalytic H_2_ production (1.5 times every year since 2004) provides clear evidences for the significance of this hot topic. Many papers studied the effect of different nanostructures and nanomaterials on the performance of photocatalysts, since their energy conversion efficiency is principally influenced by nanoscale properties.

Biomass sources have been utilized for the sustainable production of hydrogen [161,162]. A number of processes have been developed for this purpose (e.g., steam gasification [163], fast pyrolysis [162,164], and supercritical conversion [165,166]. However, these processes require harsh reaction conditions including high temperatures and/or pressures and consequently imply high costs.

Compared to these energy intensive thermochemical processes, photocatalytic reforming may be a good approach as this process can be driven by sunlight and performed at room temperature. Producing hydrogen by photocatalytic reforming of renewable biomass may also be more practical and viable than that of photocatalytic water-splitting due to its potentially higher efficiency. Water-splitting processes are relatively low efficient as limited by the recombination reaction between photogenerated electrons and holes [167].

However, to the best of our knowledge, there are only a few reports on the photocatalytic reforming of biomass to hydrogen in the literature. Pioneer studies were conducted in 1980 [168]. Kawai and Sakata reported that hydrogen could be generated from carbohydrates on RuO_2_/TiO_2_/Pt photocatalyst under 500W Xe lamp irradiation. The process is expressed as equation (1) together with the photosynthesis of carbohydrates by green plants (Equation 2), as follows:

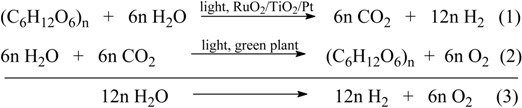

where (C_6_H_12_O_6_)_n_ represents saccharose (n = 2), starch (n ≈ 100) or cellulose (n ≈ 1,000–5,000) after hydrolysis. This method may also be applied to decomposition of excrements containing cellulose, protein and fat, acompaned by the production of H_2_ as biofuel.

The same authors subsequently reported that hydrogen could also be generated under identical conditions from other biomass sources including cellulose, dead insects, and waste materials [169,170,171]. These studies demonstrate the feasibility of the photocatalytic production of hydrogen from biomass.

Recent studies in H_2_ production from the photocatalytic reforming of glucose (a model compound of cellulose) have also been performed using M/titania catalysts (where M is Pt, Rh, Ru, Au, or Ir; titania as commercial TiO_2_ Degussa P25) [172]. Rh/TiO_2_ was found to be the optimum catalytic system providing approx. 3500 µmol of H_2_ for 0.5 g of catalyst and 5 h of irradiation.

Verykios *et al.* found that using alcohols (model compounds of biomass structure) as hole scavengers will result in increased quantum yields and enhanced rates of photocatalytic hydrogen production [173]. This is a good example of solar energy conversion into chemical energy (H_2_ as an energy carrier). The reaction is a mild 100% selective oxidation process as well as a “chemical storage of light energy”.

Hydrogen can also be photocatalytically generated from chemicals or biomass using Pt/TiO_2_ photocatalyts [174,175]. The high selectivity in this process was ascribed to the oxidation by a photoactive neutral, atomic oxygen species, detected by photoconductivity, and resulting from the neutralization of dissociatively chemisorbed O^−^(ads) species by positive photogenerated holes h^+^:

O^−^_(ads)_ + h^+^ → O*_(ads)_

### 7.2. Photo-transformation (non-catalytic) of biomass: Solar gasification

Concentrated solar energy can supply the energy to drive the gasification process [176,177,178,179]. Solar gasification decreases the amount of biomass that needs to be burned in the gasification process, thus improving the process thermal efficiency.

Heat is provided to the gasification unit using concentrated solar gasifiers and specially designed solar reactors. Two different reactor configurations are used for solar gasification including direct irradiation of the reactants through a transparent window (usually made of fused quartz) and indirect heating through an opaque wall, in which the solar energy is absorbed by a nontransparent wall and transferred to inside particles. Solar energy is also used to dry wet biomass prior to the gasification process.

**Figure 6:**
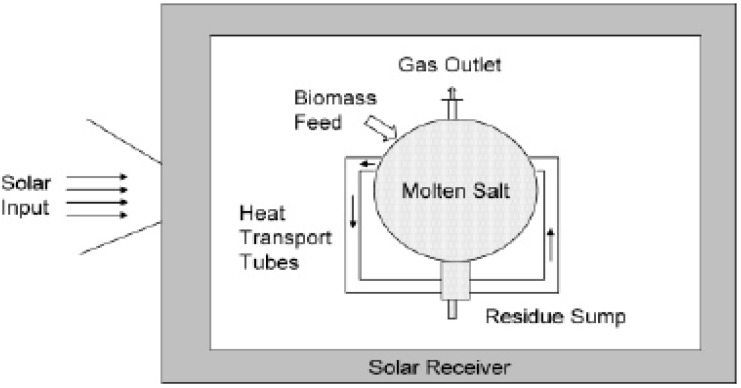
Concept of the reactor for solar gasification (Figure adapted from Adinberg *et al.* [179]. Reproduced from reference [77] by permission of the Royal Society of Chemistry.

Figure 6 shows the concept of a solar gasification reactor based on a design by Adinberg *et al.* [179]. The reactor is a central spherically or cylindrically shaped reactor. An array of vertical tubes are evenly distributed around the reactor. Incoming solar radiation is absorbed in these tubes, which contain a molten salt. The tubes provide thermal storage of the solar energy as well as a reaction chamber. Secondary concentrating optics (compound parabolic concentrator) can be added to enhance the thermal concentration and reduce thermal losses. The absorbed radiation can heat the molten salt up to approximately 850 °C.

### 7.3. Other high added-value chemicals from photochemical conversion of biomass

The photoconversion of biomass into oxygenated hydrocarbons is another interesting alternative [180,181]. The oxygen sources for this reaction can be an aqueous media and/or alcohol/CH_3_CN (more selective photo-oxidation) used to disolve sensitizing ions (e.g., Fe^3+, 2+^), or “hydrated” carbohydrates.

Selective photo-oxidation of biomass can provide a wide range of high added-value chemicals including some of the so-called platform molecules (Table 2) [182]. Platform molecules are generally compounds with various functionalities that can be turned into a plethora of chemicals and products through different catalytic transformations including oxidations, hydrogenations, amidations and esterifications [183,184,185].

**Table 2:** Top twelve sugar-derived platform molecules [182].

Platform molecule	Structure
	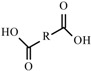
1,4-Diacids (succinic, fumaric and malic acids)	
	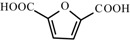
2,5-Furandicarboxylic acid	
	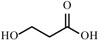
3-Hydroxypropionic acid	
	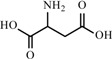
Aspartic acid	
	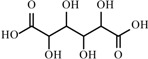
Glucaric acid	
	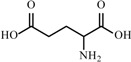
Glutamic acid	
	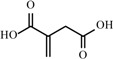
Itaconic acid	
	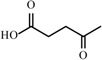
Levulinic acid	
	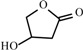
3-Hydroxybutyrolactone	
	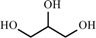
Glycerol	
	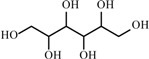
Sorbitol	
	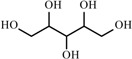
Xylitol/Arabinitol	

## 8. Future Challenges and Prospects

The main drawback of heterogeous photocatalysis (especially for degradation technology) is related to its inherently low quantum efficiency, only reaching 1% under optimised conditions (*i.e.*, only one out of one hundred incident photons is able to produce an oxidation/reduction step). Lamps can provide the necessary photons but the costs of photon production must be taken into account in the whole economics of the process and it can be very high. Sun can also give photons but only ca. 5% (ca. 30 Wm^−2^) can be used by TiO_2_, and these values are realistically insufficient.

Three main challenges exisiting in heterogeneous photochemistry need therefore to be examined so as to understand what factors govern photochemical processes in heterogeneous systems.

Firstly, identification of the factors determining the activity of photocatalysts and subsequent realisation of how these factors influence their activity. Sometimes the aim is to achieve the greatest photocatalytic activity possible, whereas in others the desire may be to completely shut down the photochemical activity of the solids` surface.

Another major and no less significant challenge is to discover how to govern the selectivity of photocatalysts and what factors manipulate this selectivity. For example, even in conventional applications of photocatalysis in water and air purification, one may often wish to achieve complete mineralization of organic pollutants without necessarily producing hazardous by-products. Of greater importance for heterogeneous photocatalysis, however, may be the photochemical synthesis of desired high-value chemicals.

The last challenge deals with efforts on how to improve the spectral sensitivity of solid metal-oxide photocatalysts so that they can absorb considerable more light energy, thus significantly improving the efficiencies in processes.

Several future trends for further development are also currently under investigation. Most of such research lines are presently at their infancies but they are envisaged to hold a great potential in the near future. These include:

- the preparation of photocatalytic nanostructures capable of selective photocatalytic degradation of organic pollutants;
- novel preparation of ternary mixed oxide systems for photooxidative degradation;
- novel photocatalyst preparations from titanium oxo families as more members of the families become available in the future;
- designing of more reliable photocatalyst that can be photoactivated by visible and/or solar light;
- exploring the possibilities to work with other materials than titania (e.g., metal sulfides);
- photosensitizing TiO_2_ in the visible by doping, especially by platinization and continue the investigation of anionic doping;
- taking advance of photocatalysis for preparative fine chemistry;
- use of photocatalysis as a new medical tool (e.g., in cancer treatment);
- novel photocatalysts for the production of energy: biohydrogen either from biomass or from photocatalytic spliting of water.
